# HBO1 overexpression is important for hepatocellular carcinoma cell growth

**DOI:** 10.1038/s41419-021-03818-1

**Published:** 2021-05-26

**Authors:** Wenhui Zhong, Heping Liu, Li Deng, Guohua Chen, Yubin Liu

**Affiliations:** 1 Department of Hepatobiliary Surgery, Guangdong Provincial People’s Hospital, Guangdong Academy of Medical Sciences, Guangzhou, China; 2 Guangzhou Yiyang Bio-technology Co. Ltd, Guangzhou, China; 3 Guangzhou Beogene Biotech, Guangzhou, China

**Keywords:** Targeted therapies, Oncogenes

## Abstract

Hepatocellular carcinoma (HCC) is a common primary liver malignancy lacking effective molecularly-targeted therapies. HBO1 (lysine acetyltransferase 7/KAT7) is a member of MYST histone acetyltransferase family. Its expression and potential function in HCC are studied. We show that *HBO1* mRNA and protein expression is elevated in human HCC tissues and HCC cells. HBO1 expression is however low in cancer-surrounding normal liver tissues and hepatocytes. In HepG2 and primary human HCC cells, shRNA-induced HBO1 silencing or CRISPR/Cas9-induced HBO1 knockout potently inhibited cell viability, proliferation, migration, and invasion, while provoking mitochondrial depolarization and apoptosis induction. Conversely, ectopic overexpression of HBO1 by a lentiviral construct augmented HCC cell proliferation, migration and invasion. In vivo, xenografts-bearing HBO1-KO HCC cells grew significantly slower than xenografts with control HCC cells in severe combined immunodeficient mice. These results suggest HBO1 overexpression is important for HCC cell progression.

## Introduction

Liver cancer is the fifth most common cancer and the 2th leading cause of cancer-related human mortalities globally, with over 840,000 new cases and 780,000 deaths reported each year^1,2^. Of which 75–80% of liver cancer is hepatocellular carcinoma (HCC), the latter is considered as a major health threat globally^3,4^. The overall five-year survival of HCC is far more from satisfactory^5^. The current treatments for HCC are limited, and sorafenib is the only molecularly targeted agent^3,4^. Therefore, patients with advanced HCCs, including those with unresectable, recurrent, and metastatic HCCs, often have very poor prognosis^3,4^. In the past years, HCC’s incidence has been steadily rising, especially in Western countries^3,4^. Thus, it is extremely urgent to explore and develop novel anti-HCC strategies and molecularly-targeted agents^6,7^.

Acetylation is an universal protein modification vital for almost all cellular behaviors, from cell cycle progression, gene transcription and expression, signaling transduction, RNA splicing and cellular metabolism, among others^8,9^. The histone acetylation will lead to chromatin unfold, which facilitates proteins accessing DNA being replicated and/or transcribed^10,11^. Histone acetylation on lysine residues is a reversible step regulated by the antagonistic actions of two enzymes: histone acetyltransferases and histone deacetylases^12^. Dysregulation of histone acetylation is commonly detected in HCC, which is associated with HCC tumorigenesis, progression, and therapy resistance^13^. However, the underlying mechanisms are still largely unknown.

The multifunctional HBO1 (lysine acetyltransferase 7/KAT7) is a primary and essential member of MYST (MOZ, Ybf1/Sas3, Sas2, and Tip60) family histone acetyltransferases^10^. It is responsible for the acetylation of histone H4 and H3K14^10^. HBO1 is vital for the pre-replication complex (pre-RC) formation, DNA replication, and cell proliferation via acetylation of histone H4 and H3^14^. HBO1-dictated H3K14ac initiates de novo activation of key embryonic patterning genes during embryonic development^15,16^. HBO1 is actively involved in regulating multiple and key cellular and physiological functions, including DNA replication, gene transcription, and protein ubiquitination as well as immune regulation, stem cell pluripotent, self-renewal maintenance, and embryonic development^10,17,18^.

HBO1 associates with chromatin licensing and DNA replication factor 1 and is required for G1 phase cell cycle progression^16,19^. In addition, HBO1 functions as a positive regulator of centromeric CENPA (Centromere Protein A) by preventing SUV39H1-mediated centromere inactivation^20^. MacPherson et al., discovered that HBO1 is essential for the acetylation of H3K14 (H3K14ac), thereby promoting the processivity of RNA polymerase II to maintain high expression of key oncogenic genes (*MYLK*, *HOXA9, HOXA10*, and several others) in leukemia stem cells^21^.

Recent studies have implied an essential role of HBO1 in cancer cell progression^22^. HBO1 silencing by targeted short hairpin RNA (shRNA) resulted in significant proliferation inhibition in MCF7 and HeLa cancer cell lines^14,15^. HBO1 activated Wnt/β-catenin signaling to assist bladder cancer cell proliferation^23^. Taniue et al., found that HBO1-mediated downregulation of tumor suppressor candidate 3 (TUSC3) is essential for colon cancer cell proliferation^24^. Kueh and colleagues however showed that HBO1 did not have an essential role in cell proliferation and DNA replication in HEK293T, MCF7, and HeLa cancer cell lines^25^. Expression and potential function of HBO1 in HCC have not been studied thus far. In the present study, we show that HBO1 overexpression is important for HCC cell growth.

## Materials and methods

### Chemicals and reagents

Antibodies of HBO1 (#58418), cleaved caspase-3 (#9664), acetyl-Histone H3 at Lys14 (H3K14, #9927), Histone H3 (#9927), cleaved-poly (ADP-ribose) polymerase (PARP) (#5625), cleaved-caspase-9 (#20750), β-catenin (#8480), β-actin (#4970), and Tubulin (#2125) were obtained from Cell Signaling Tech China (Shanghai, China). The cell culture reagents were obtained from Gibco Co. (Beijing, China).

### Cell culture

HepG2 HCC cell line and the HL-7702 hepatocytes were obtained from the Cell Bank of CAS Shanghai (Shanghai, China) and cultured as described^26^. The primary human HCC cells, derived from five primary HCC patients, HCC-1/HCC-2/HCC-3/HCC-4/HCC-5, were provided by Dr. Lu at Nanjing Medical University^26,27^. The primary human HCC cells were cultured as described^26^. Human primary adult hepatocytes, purchased from the Cell Bank of Fudan University (Shanghai, China), were derived from the liver of a partial hepatectomy patient. Human hepatocytes were cultured in primary cell culture medium as described previously^26^. In the present studies, protocols testing human tissues and primary human cells were approved by Ethics Review Board of Guangdong Academy of Medical Sciences. The written-informed consent was obtained from each primary HCC patient. All studies were conducted according to the principles expressed in the Declaration of Helsinki and international guidelines.

### Human tissues

Human HCC tissues and the surrounding normal liver tissues were from 10 individual written-informed primary HCC patients, enrolled at Guangdong Provincial People’s Hospital. HCC patients enrolled received no chemotherapy and radiotherapy before surgery. Tissues were incubated with the described lysis buffer (Biyuntian, Wuxi, China) and tissue lysates stored in liquid nitrogen. The written-informed consent was obtained from each participant. The protocols of this study were approved by the Ethic Committee of Guangdong Provincial People’s Hospital, Guangdong Academy of Medical Sciences.

### Quantitative real-time PCR (qPCR)

Total RNA was extracted by Trizol reagents, reverse-transcribed and qPCR were performed using the SYBR green kit by an ABI-7500 system (Applied Biosystems, Shanghai, China)^28^. For data quantification, a 2^ΔΔCt^ method was utilized, and *GAPDH* tested as the reference gene and internal control. All the primers were synthesized by Genechem (Shanghai, China).

### HBO1 shRNA

A pair of lentiviral GV369 constructs (containing GFP gene and puromycin selection gene), encoding non-overlapping HBO1 shRNA sequences, namely HBO1-shRNA-1/2, were designed, synthesized, and verified by Shanghai Genechem (Shanghai). The construct together with the lentivirus-packing plasmids (psPAX2 and pMD2.G, Shanghai Genechem Co.) were co-transfected to HEK-293T cells, generating HBO1-shRNA lentivirus. The viruses were added to human HCC cells (cultured into six-well tissue plates at 2 × 10^5^ cells per well). After 24 h, virus-containing medium was replaced with fresh complete medium, and cells were subjected to FASC sorting to generate monoclonal cells (GFP-positive). Stable HCC cells were further selected by puromycin (5 μg/mL, Sigma) for 10 days. HBO1 silencing in stable cells was verified by qPCR and Western blotting assays.

### HBO1 knockout

The small guide RNA (sgRNA) targeting human *HBO1* (Target DNA Sequence: GATGAACGAGTCTGCCGAAG. PAM Sequence: AGG) was inserted into a lenti-CRISPR-GFP-puro plasmid (from Dr. Chen at Jiangsu University^29^). The construct was transfected to HCC cells by using Lipofectamine 2000. Afterwards, GFP-positive cells were sorted by FACS and resulting monoclonal cells were selected by puromycin (5 μg/mL)-containing medium. HBO1 knockout in stable cells was screened by qPCR and Western blotting assays.

### Western blotting

In brief, the protein lysates, from human tissues or cultured cells, were separated by 10–12% SDS-PAGE gels (40 μg protein in each lane), and transferred to polyvinylidene difluoride (PVDF) blots (EMD Millipore, Shanghai, China). The blots were blocked and incubated with the applied primary and secondary antibodies, with antibody–antigen binding examined by an ECL kit (GE Healthcare, Chicago, IL, USA). The same set of lysates were run in sister gels to test different proteins. The ImageJ software was utilized for data quantification.

### Cell-counting kit 8 (CCK-8) assay

Cells were trypsinized and inoculated into the 96-well tissue-culture plates at 3500 cells per well. After incubation at 37 °C for 96 h, 10 µL of CCK-8 reagent (Dojindo, Kumamoto, Japan) was added into each well for 2 h. CCK-8 absorbance, the optical density (OD), was always examined at 450 nm.

### Colony formation

HCC-1 primary cells, with applied genetic modifications, were initially seeded at 10,000 cells per well into 10-cm tissue-culture plates. The complete medium was renewed every two days (total culture for 10 days), and large colonies (>100 cells/per colony) stained and manually counted.

### Migration and invasion assays

The established and primary human HCC cells were trypsinized and suspended into serum-free medium. “Transwell” chambers with 8 μm pore-size were utilized (BD Biosciences, Shanghai, China). For each condition, 30,000 cells were added to the upper surface of the chamber, with the lower chamber filled with complete medium (10% FBS). Cells were allowed to migrate for 16 h, excluding the possible influence from proliferation/viability change. Afterwards, the migrated cells, in the lower chamber, were fixed, stained and counted. For the invasion assays, “Matrigel” (Sigma, Shanghai, China) was coated to the “Transwell” chambers.

### EdU (5-ethynyl-20-deoxyuridine) staining

The established or primary human HCC cells, with or without the applied genetic modifications, were seeded into twelve-well tissue culture plates (at 0 × 10^5^ cells per well), cells were cultured for 72 h. An EdU Apollo-567 kit (RiboBio, Guangzhou, China) was utilized, and the cell proliferation ratio (EdU/DAPI×100%) calculated from at least 500 nuclei in five random views per treatment.

### Annexin V fluorescence activated cell sorting (FACS)

HCC cells, with the applied genetic modifications, were seeded into six-well tissue culture plates (at 2 × 10^5^ cells per well), cells were cultured for 48 h and stained with Annexin V-FITC and propidium Iodide (PI) (each at 10 μg/mL). Cells were then subjected to flow cytometry (Beckman Coulter, Brea, CA). The Annexin V-positive cells were gated, and its ratio recorded.

### TUNEL assay

HCC cells, with the applied genetic modifications, were seeded into 24-well tissue culture plates (at 0.3 × 10^5^ cells per well), cells were further cultured for 48 h and incubated with TUNEL (Invitrogen) for 3 h and DAPI for 5 min. TUNEL and DAPI staining was visualized under a fluorescent microscope (Leica). TUNEL ratio (TUNEL/DAPI×100%) was calculated from at least 500 nuclei in five random views per treatment.

### Caspase-3/Caspase-9 activity assay

As described^30^, HCC cells, with or without the applied genetic modifications, were seeded into six-well tissue culture plates (at 2 × 10^5^ cells per well), cells were cultured for 48 h. For each treatment 20 μg of cytosolic extracts were mixed with the caspase assay buffer^30^ along with 7-amido-4-(trifluoromethyl)-coumarin (AFC)-conjugated caspase-3/caspase-9 substrate^30^. AFC optic density (OD) was examined by a Fluoroskan system^30^.

### Mitochondrial depolarization

In stressed cells with mitochondrial depolarization, the JC-1 fluorescence dye can aggregate in mitochondria, forming green JC-1 monomers^31^. HCC cells, with or without the applied genetic modifications, were seeded into 24-well tissue culture plates (at 0.2 × 10^5^ cells per well), cells were further cultured for 48 h and incubated with JC-1 (5 μg/mL) for 30 min under the dark at room temperature. Afterwards, cells were washed and tested under a fluorescence spectrofluorometer at 488 nm. The representative JC-1 images, merging both green and red fluorescence channels, were presented as well.

### Ectopic HBO1 overexpression

A GV369 construct with HBO1-cDNA was designed, synthesized and verified by Shanghai Genechem (Shanghai, China). The construct together with the lentivirus-packing plasmids were co-transfected to HEK-293T cells, generating HBO1 expression lentivirus. HCC-1 cells were transfected with the viruses for 12 h, and the virus-containing medium was then replaced with fresh complete medium. Cells were subjected to FASC sorting to generate monoclonal cells (GFP-positive cells). Stable cells were selected by puromycin, and two stable cell lines were established. HBO1 overexpression was verified by qPCR and Western blotting analyses.

### Tumor growth in vivo

SCID mice (half male half female, 5–6 week old) were purchased from the Experimental Animal Center, School of Medicine of Zhejiang University (Hangzhou, China), maintained under specific pathogen-free (SPF) conditions. All animal studies in the present study are in accordance with IACUC as well as international guidelines and regulations, with the protocols approved by Ethics Review Board of Guangdong Academy of Medical Sciences. The exponentially growing HCC-1 primary cells (1 × 10^7^ cells per mice), with CRISPR/Cas9 HBO1-KO construct or the empty vector, were subcutaneously injected into the loose skin in the right front leg of the recipient SCID mice. Within 2 weeks the xenograft HCC-1 tumors were established and recordings were started. The animal studies were approved by Institutional Animal Care and Use Committee (IACUC) and Ethics Committee of Guangdong Provincial People’s Hospital, Guangdong Academy of Medical Sciences. Tumors were cut into small pieces and lysed in tissue lysis buffer (Biyuntian, Wuxi, China). Protein lysates were tested by Western blotting^32^.

### Statistical analysis

In vitro experiments were repeated at least three times and similar results were obtained. Quantitative results were expressed as mean ± standard deviation (SD). Statistical analyses among different groups were performed by one-way ANOVA with Scheffe’s test using SPSS23.0 software (SPSS Inc., Chicago, IL). The two-tailed unpaired *T*-test (Excel 2007) was utilized when testing the significance between two treatment groups. *P* values of <0.05 were considered statistically significant.

## Results

### HBO1 is upregulated in human HCC tissues and cells

**Fig. 1:**
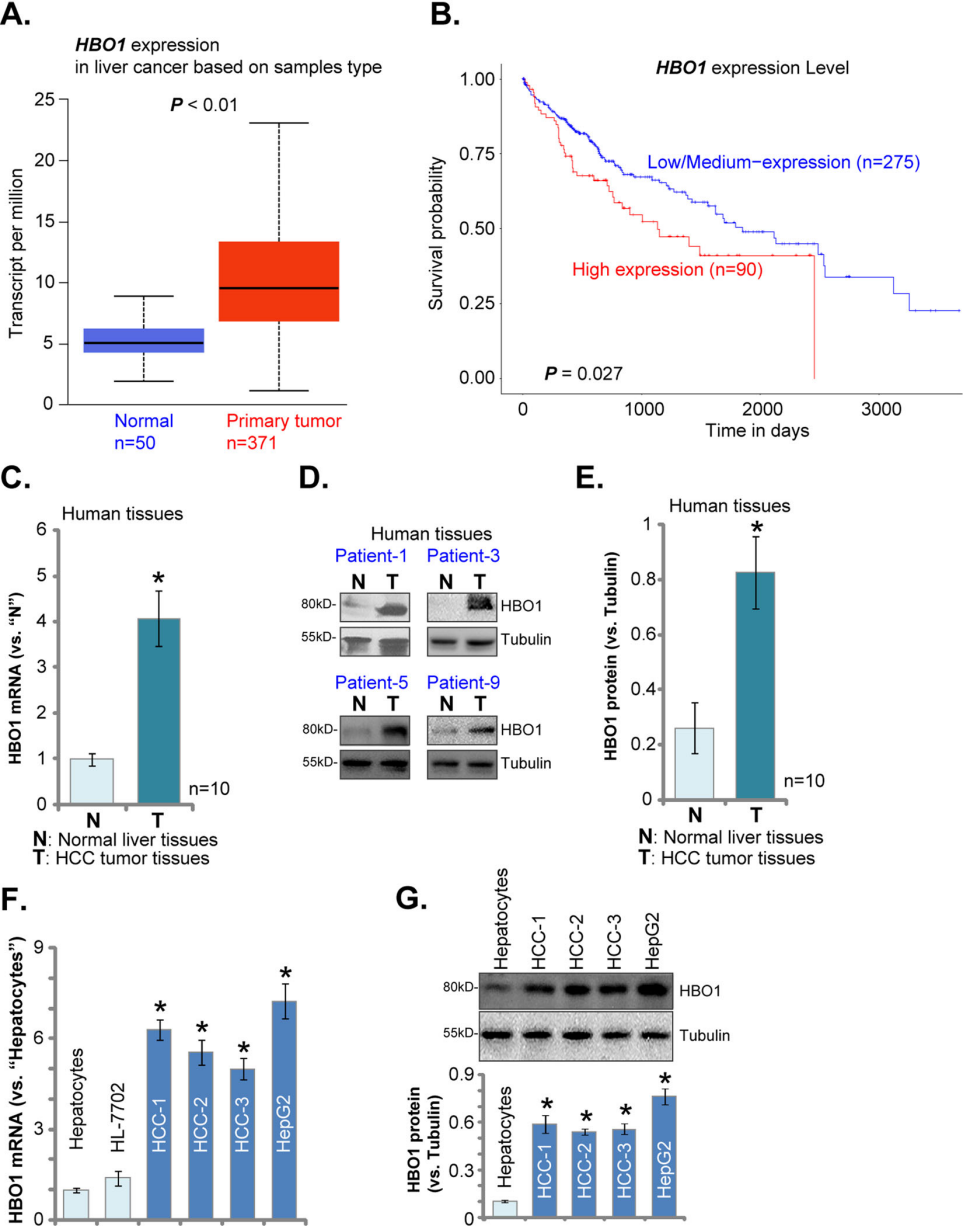
HBO1 is upregulated in human HCC tissues and cells. UALCAN database shows *HBO1* expression (Transcript per million) in 371 cases of liver cancer tissues and 50 cases of normal adjacent liver tissues (**A**). Kaplan–Meier survival analyses of HBO1-Low/Medium (*n* = 275) and HBO1-High (*n* = 90) liver cancer patients (**B**). Expression of *HBO1* mRNA (**C**) and protein (**D**, **E**) in fresh tissue lysates of ten (*n* = 10) pairs of human HCC tissues (“T”) and matched surrounding normal liver tissues (“N”) was shown. Expression of *HBO1* mRNA (**F**) and protein (**G**) in the listed hepatocytes, HL-7702 cells and HCC cells was shown. Error bars indicate mean ± standard deviation (SD, *n* = 5). *P < 0.05 vs. “N” tissues/“Hepatocytes” (**C**–**G**). The experiments in this figure were repeated five times, and similar results were obtained.

First, we tested the expression of HBO1 in human HCC. UALCAN is a comprehensive and interactive web resource for analyzing cancer data. It performs in-depth analysis of gene expression data in different cancer databases, including TCGA, MET500, and CPTAC. It also provides graphs and plots depicting expression profile and patient survival information for protein-coding genes^33^. Through searching the UALCAN database (http://ualcan.path.uab.edu), we found that *HBO1* mRNA expression in liver cancer tissue specimens is significantly higher than that in normal liver tissues (*P* < 0.01, Fig. 1A). Furthermore, *HBO1* overexpression is associated with poor overall-survival in liver cancer patients (*P* = 0.027 Fig. 1B).

To verify the bioinformatics results, HCC tissues, derived from ten human patients with primary HCC, as well as the matched surrounding normal liver tissues, were obtained. Tissue lysates were achieved by incubation tissues with the tissue lysis buffer. Testing mRNA expression, using qPCR assays, show that *HBO1* mRNA expression in HCC tissues (“T”) is over four folds of that in the surrounding normal liver tissues (“N”) (Fig. 1C). Western blotting assay results, Fig. 1D, confirmed HBO1 protein upregulation in HCC tissues of representative human patients, while its expression is relatively low in matched surrounding normal liver tissues (Fig. 1D). Quantitative analyses of HBO1 protein expression from all ten pairs of tissues confirmed that HBO1 protein upregulation in HCC tissues is significant (*P* < 0.05 vs. normal liver tissues) (Fig. 1E).

Further studies were performed to test HBO1 expression in human HCC cells. Primary human HCC cells, derived from three primary HCC patients, HCC-1/HCC-2/HCC-3 (from Dr. Lu at Nanjing Medical University^26,27^), were tested. Results in Fig. 1F demonstrated that *HBO1* mRNA is elevated in primary human HCC cells and established HepG2 cells. Conversely, *HBO1* mRNA expression is relatively low in primary human hepatocytes and HL-7702 hepatocytes^26,27^ (Fig. 1F). HBO1 protein expression was elevated in HepG2 and primary human HCC cells as well (Fig. 1G). Yet, a low HBO1 protein expression is detected in primary human hepatocytes (Fig. 1G). These results imply that HBO1 is upregulated in human HCC tissues and cells.

### HBO1 shRNA inhibits HCC cell viability, proliferation, migration, and invasion

**Fig. 2:**
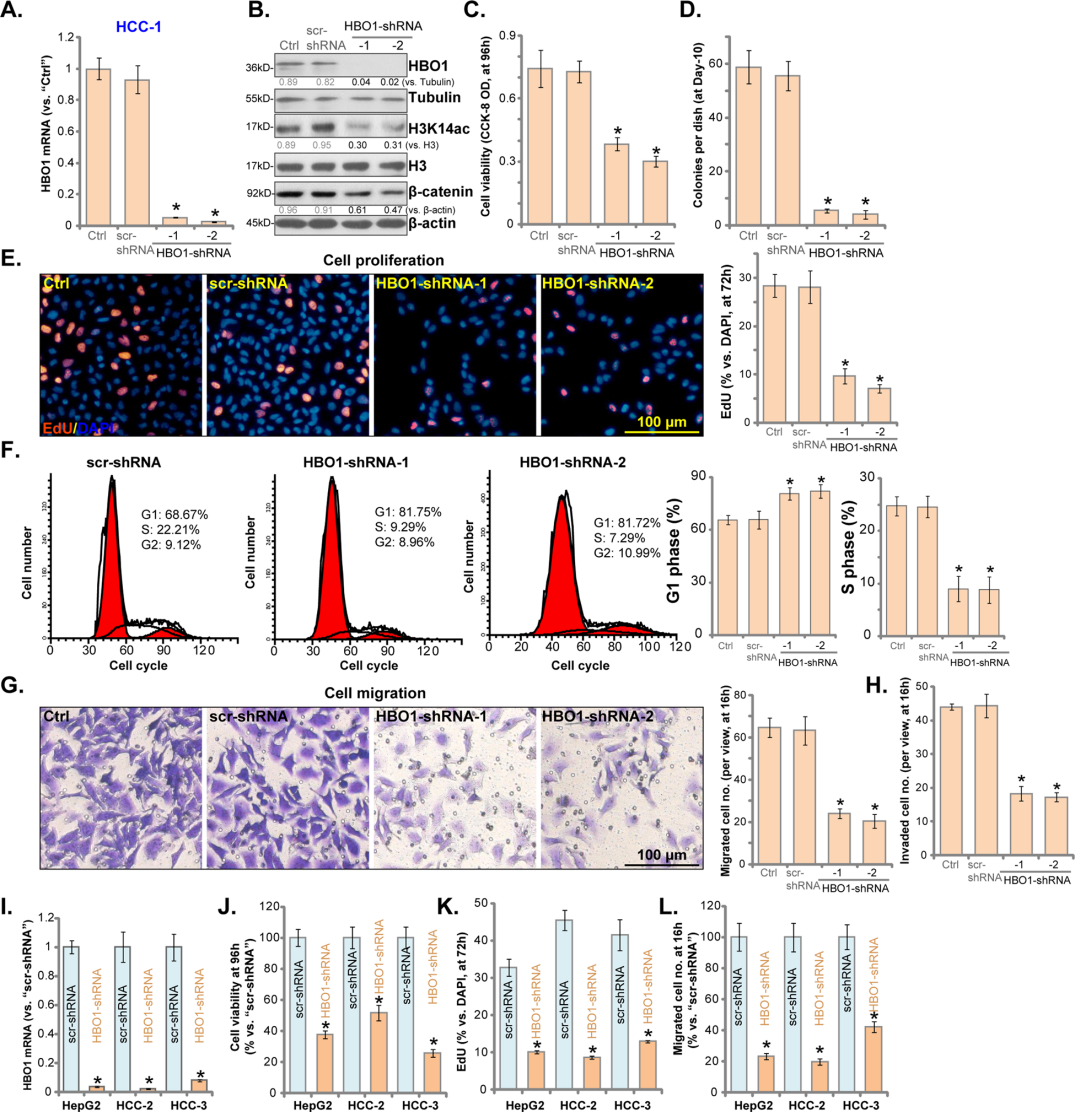
HBO1 shRNA inhibits HCC cell viability, proliferation, migration and invasion. Stable primary human HCC cells (HCC-1/HCC-2/HCC-3) and HepG2 cells, with scramble control shRNA (“scr-shRNA”) or the applied HBO1 shRNA (“−1/−2”, with non-overlapping sequences), as well as the parental control HCC cells (“Ctrl”), were cultured, expression of *HBO1* mRNA (**A**, **I**) and protein (**B**) was shown; Cellular functions, including cell viability (CCK-8 OD, **C**, **J**), proliferation (colony formation and nuclear EdU incorporation, **D**, **E**, **K**), cell cycle progression (**F**), cell migration and invasion (**G**, **H**, **L**), were tested, with results quantified. Expression of listed proteins was quantified and normalized to the loading control (**B**). Error bars indicate mean ± standard deviation (SD, *n* = 5). *P < 0.05 vs. “Ctrl” cells or “scr-shRNA” cells. The experiments in this figure were repeated five times, and similar results were obtained. Scale bar = 100 μm (**E**, **G**).

To study the potential function of HBO1 in HCC cells, two lentiviral GV369 constructs (with GFP and puromycin selection gene), encoding non-overlapping sequences of HBO1 shRNA (HBO1-shRNA-1/2^21^), were generated. The two were individually transduced to HCC-1 primary cells (see Fig. 1). Following GFP sorting (FACS) and puromycin selection, stable HCC-1 cells were established. Testing mRNA expression, using qPCR analyses, demonstrated that *HBO1* mRNA levels decreased over 95% in stable HCC-1 cells with HBO1 shRNA (vs. control cells, Fig. 2A). Consequently, HBO1 protein levels were significantly downregulated (Fig. 2B). HBO1 shRNA significantly inhibited H3K14 acetylation (Fig. 2B).

Two groups have performed RNA-Seq studies to test differentially-regulated mRNAs in control cells versus HBO1-depleted cells^21,34^. A number of these HBO1-regulated genes play oncogenic roles in HCC, including *C‑C chemokine receptor type 2* (*CCR2*)^35,36^, *myosin light chain kinase* (*MYLK*)^37^, *VEGFR2*^38,39^, *Homeobox A10* (*HOXA10*)^40,41^, *pre-leukemia transcription factor 3* (*PBX3*)^42,43^ and *frizzled-related protein* (*FRZB*)^44^. We found that shRNA-induced silencing of HBO1 downregulated *MYLK*, *VEGFR2*, *PBX3*, *CCR2*, *HOXA10* and *FRZB* mRNAs (Fig. S1A) in HCC-1 cells. Furthermore, HBO1 was reported to activate Wnt/β-catenin signaling pathway and to promote upregulation and nuclear localization of β-catenin in cancer cells^23^. We found that β-catenin protein levels were decreased in HCC-1 cells with HBO1 silencing (Fig. 2B).

To study the functional results of HBO1 silencing, a CCK-8 viability assay was performed. As shown, HBO1 silencing resulted in significant viability reduction in HCC-1 cells (Fig. 2C). Furthermore, in HCC-1 cells HBO1 shRNA largely inhibited colony formation (Fig. 2D) and nuclear EdU incorporation (Fig. 2E), indicating proliferation inhibition. Cell cycle progression assessed by flow cytometry with propidium iodine (PI) showed that HBO1 shRNA induced an increase in the G1 cell population with a concomitant decrease of the cell population in S phase in HCC-1 cells. Thus, HBO1 silencing induced G1-S cell cycle arrest in HCC-1 cells (Fig. 2F). Additionally, HBO1-silenced HCC-1 cells showed significantly inhibited cell migration and invasion, which were tested by “Transwell” assay (Fig. 2G) and “Matrigel Transwell” assay (Fig. 2H), respectively.

To study the potential function of HBO1 in other HCC cells, HepG2 cells and primary human HCC cells-derived from two other HCC patients (“HCC-2/HCC-3”) were transduced with the lentiviral HBO1-shRNA-2. Stable cells were established with GFP sorting and puromycin selection. As shown in stable HCC cells with HBO1-shRNA, *HBO1* mRNA levels downregulated over 90% (Fig. 2I). In the HCC cells, HBO1 shRNA resulted in robust viability (CCK-8 OD) reduction (Fig. 2J), proliferation inhibition (reduced nuclear EdU ratio, Fig. 2K) and cell migration decrease (results quantified in Fig. 2L). The scramble control shRNA, scr-shRNA, exerted no significant effect on HBO1 expression (Fig. 2A, B) and functions of HCC cells (Fig. 2C–L).

In the primary human hepatocytes, infection with the HBO1–shRNA-2 lentivirus resulted in potent *HBO1* mRNA reduction (Fig. S2A). However, HBO1 silencing failed to inhibit cell viability (Fig. S2B) and proliferation (Fig. S2C) in hepatocytes, indicating a specific effect in the cancerous cells. Collectively, these results show that HBO1 silencing inhibited HCC cell viability, proliferation, migration, and invasion.

### HBO1 silencing provokes apoptosis in HCC cells

RNA-seq microarray analysis in HBO1-depleted cells identified over 250 differentially regulated genes. A number of them are anti-apoptosis genes^34^. Furthermore, recent studies have reported apoptosis activation in cells with HBO1 depletion^21^. CRISPR/Cas9-induced HBO1 KO was shown to induce apoptosis activation in leukemia stem cells^21^. Similarly, in AML cells HBO1 depletion by CRISPR/Cas9 method reduced proliferation and increased apoptosis activation^45^.

**Fig. 3:**
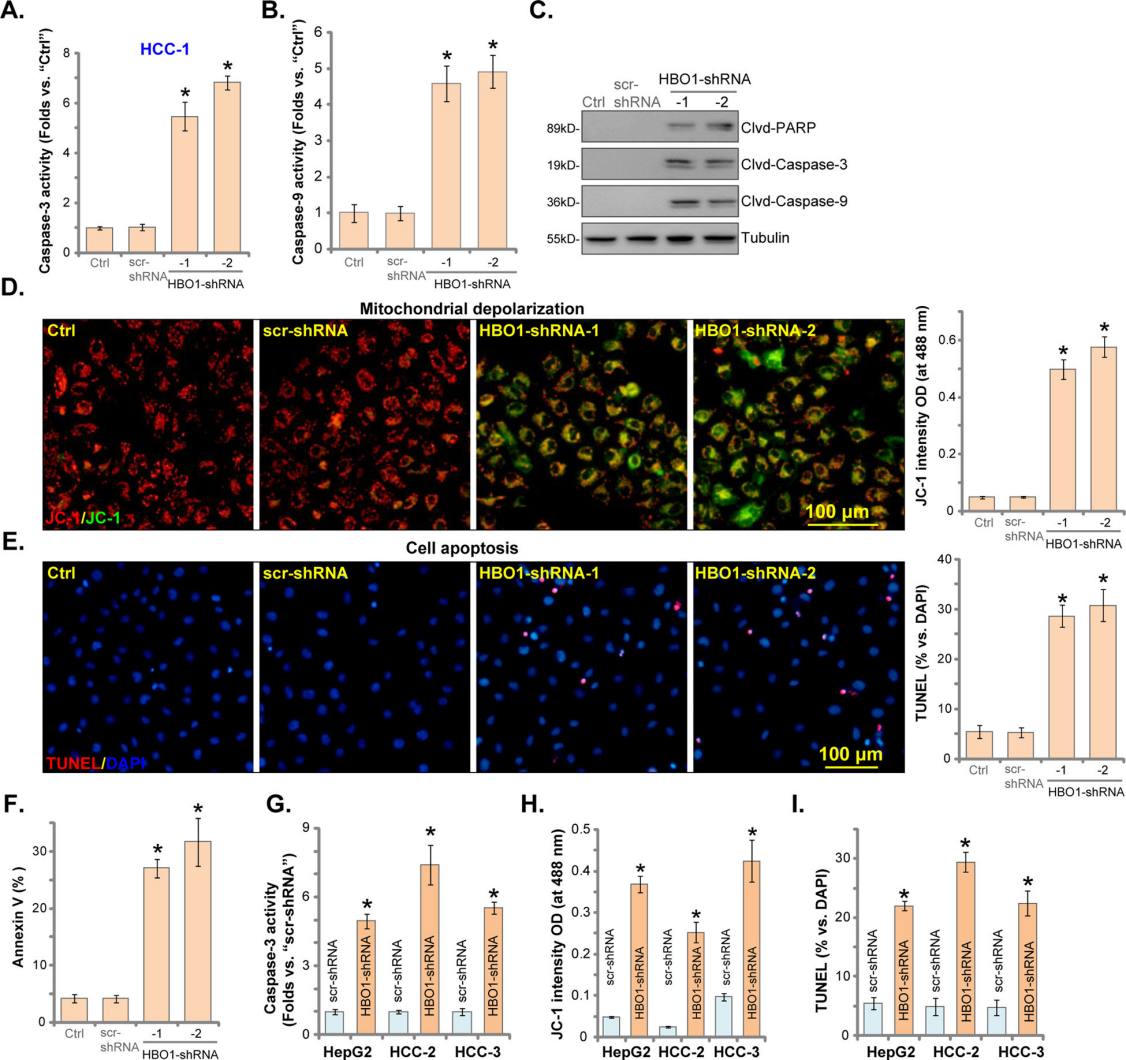
HBO1 silencing provokes apoptosis in HCC cells. Stable primary human HCC cells (HCC-1/HCC-2/HCC-3) and HepG2 cells, with scramble control shRNA (“scr-shRNA”) or the applied HBO1 shRNA (“−1/−2”, with non-overlapping sequences), as well as the parental control HCC cells (“Ctrl”), were cultured for 48 h, the relative caspase-3/caspase-9 activity (**A**, **B**, **G**), expression of apoptosis-associated proteins (**C**), mitochondrial depolarization (JC-1 green monomers intensity assays, **D**, **H**) were tested; Cell apoptosis was tested by nuclear TUNEL staining (**E**, **I**) and Annexin V FACS (**F**), with results quantified. Error bars indicate mean ± standard deviation (SD, *n* = 5). *P < 0.05 vs. “Ctrl” cells or “scr-shRNA” cells. The experiments in this figure were repeated five times, and similar results were obtained. Scale bar = 100 μm (**D**, **E**).

We next studied whether HBO1 silencing can provoke apoptosis in HCC cells. As demonstrated, in stable HCC-1 cells with HBO1 shRNA (HBO1-shRNA-1/2, see Fig. 2), activities of caspase-3 (Fig. 3A) and caspase-9 (Fig. 3B) were significantly elevated. Cleavages of caspase-3, caspase-9 and PARP (poly-ADP ribose polymerase) were detected in HBO1 shRNA-expressing HCC-1 cells as well (Fig. 3C). Furthermore, HBO1 silencing in HCC-1 cells resulted in mitochondrial depolarization, the latter was evidenced by JC-1 green monomers accumulation in mitochondria^31^ (Fig. 3D). To confirm apoptosis activation we show that nuclear TUNEL ratio (Fig. 3E) and Annexin-positive staining (Fig. 3F) were both significantly elevated in HBO1 shRNA-expressing HCC-1 cells.

In HepG2 cells and primary HCC cells (derived from two other HCC patients, HCC-2/HCC-3), HBO1 silencing by HBO1-shRNA-2 (see Fig. 2) increased caspase-3 activity (Fig. 3G) and induced mitochondrial depolarization (JC-1 green monomers intensity increase, Fig. 3H). To confirm cell apoptosis, nuclear TUNEL ratio was significantly increased in HBO1-silenced HCC cells (Fig. 3I). These results show that HBO1 shRNA provoked apoptosis in human HCC cells. The scramble control shRNA, scr-shRNA, failed to induce significant apoptosis activation in HCC cells (Fig. 3A–I). Notably, in the primary human hepatocytes, HBO1 silencing by targeted shRNA failed induce significant apoptosis activation (TUNEL assay, Fig. S2D).

### HBO1 knockout inhibits HCC cell progression in vitro

**Fig. 4:**
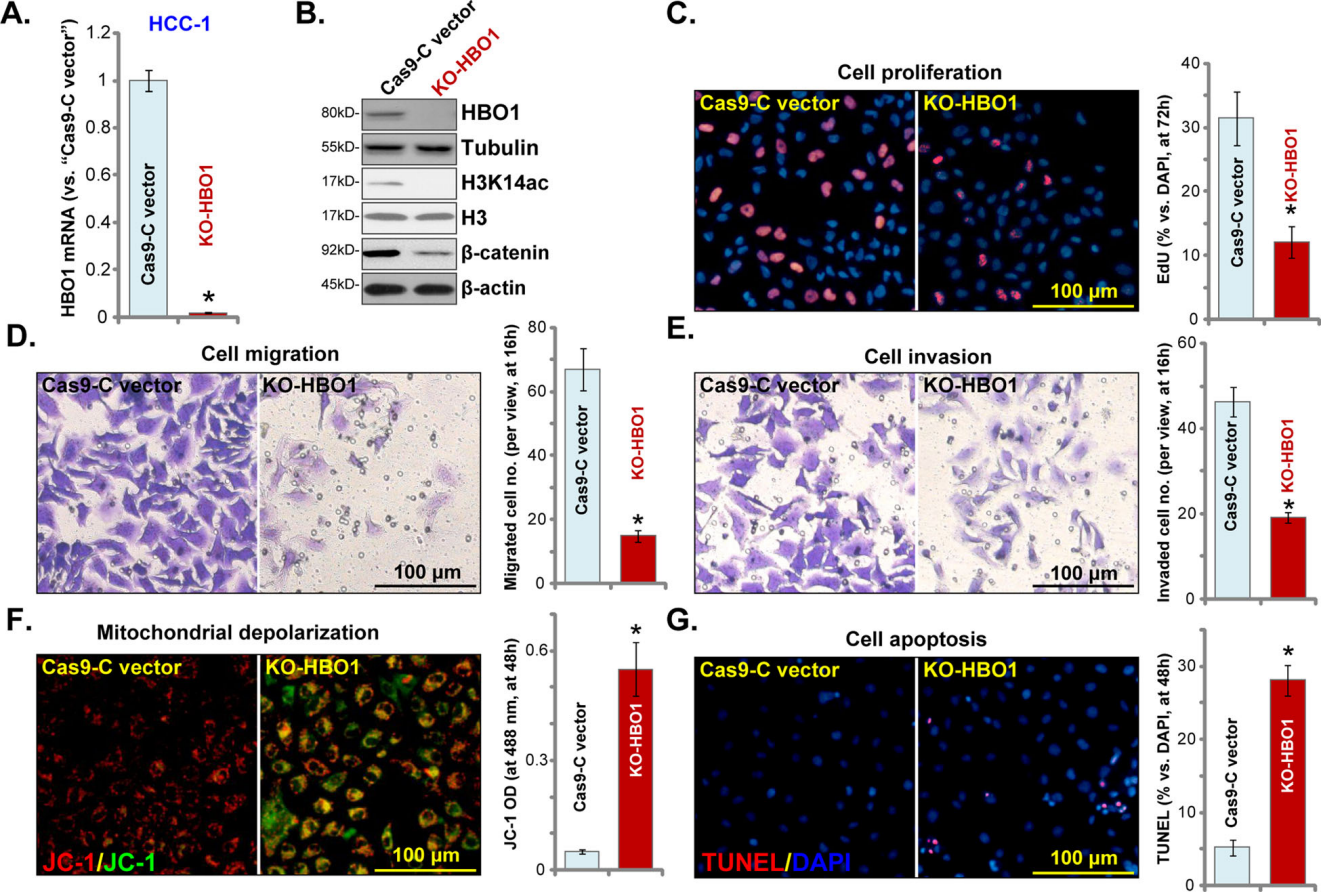
HBO1 knockout inhibits HCC cell progression in vitro. Stable primary human HCC cells, HCC-1, with the CRISPR/Cas9 HBO1-KO construct (“KO-HBO1”) or empty vector (“Cas9-C vector”) were cultured for applied time periods, expression of *HBO1* mRNA (**A**) and protein (**B**) was shown; Cell proliferation (nuclear EdU incorporation, **C**), cell migration and invasion (**D**, **E**), as well as mitochondrial depolarization (JC-1 green monomers accumulation, **F**) and cell apoptosis (nuclear TUNEL staining, **G**) were tested, with results quantified. Error bars indicate mean ± standard deviation (SD, *n* = 5). *P < 0.05 vs. “Cas9-C vector” cells. The experiments in this figure were repeated five times, and similar results were obtained. Bar = 100 μm (**C**–**G**).

To exclude the possible off-target effect of the applied HBO1 shRNA, a CRISPR/Cas9 vector containing HBO1 sgRNA was constructed and transduced to HCC-1 primary cells. Stable monoclonal cells, KO-HBO1 cells, were established with FACS-mediated GFP sorting and puromycin selection. As compared to the control cells with CRISPR/Cas9 empty vector (“Cas9-C vector”), *HBO1* mRNA (Fig. 4A), and protein (Fig. 4B) expression was almost completely depleted in KO-HBO1 HCC-1 cells. H3K14 acetylation was almost abolished (Fig. 4B). In KO-HBO1 HCC-1 cells, HBO1-regulated mRNAs, including *MYLK*, *VEGFR2*, *PBX3*, *CCR2*, *HOXA10,* and *FRZB*^21,34^, were dramatically downregulated (Fig. S1B), β-catenin protein levels were decreased as well (Fig. 4B).

Functional studies demonstrated that CRISPR/Cas9-induced HBO1 KO largely inhibited HCC-1 cell proliferation, evidenced by decreased nuclear EdU staining (Fig. 4C). Furthermore, in vitro cell migration (Fig. 4D) and invasion (Fig. 4E) were largely inhibited in KO-HBO1 HCC-1 cells. Significantly, HBO1 KO resulted in mitochondrial depolarization (JC-1 green monomers accumulation, Fig. 4F) and cell apoptosis (increase of TUNEL ratio, Fig. 4G). Therefore, CRISPR/Cas9-induced HBO1 KO inhibited HCC cell proliferation, migration and invasion, but inducing apoptosis activation.

### Ectopic overexpression of HBO1 promotes HCC cell progression in vitro

**Fig. 5:**
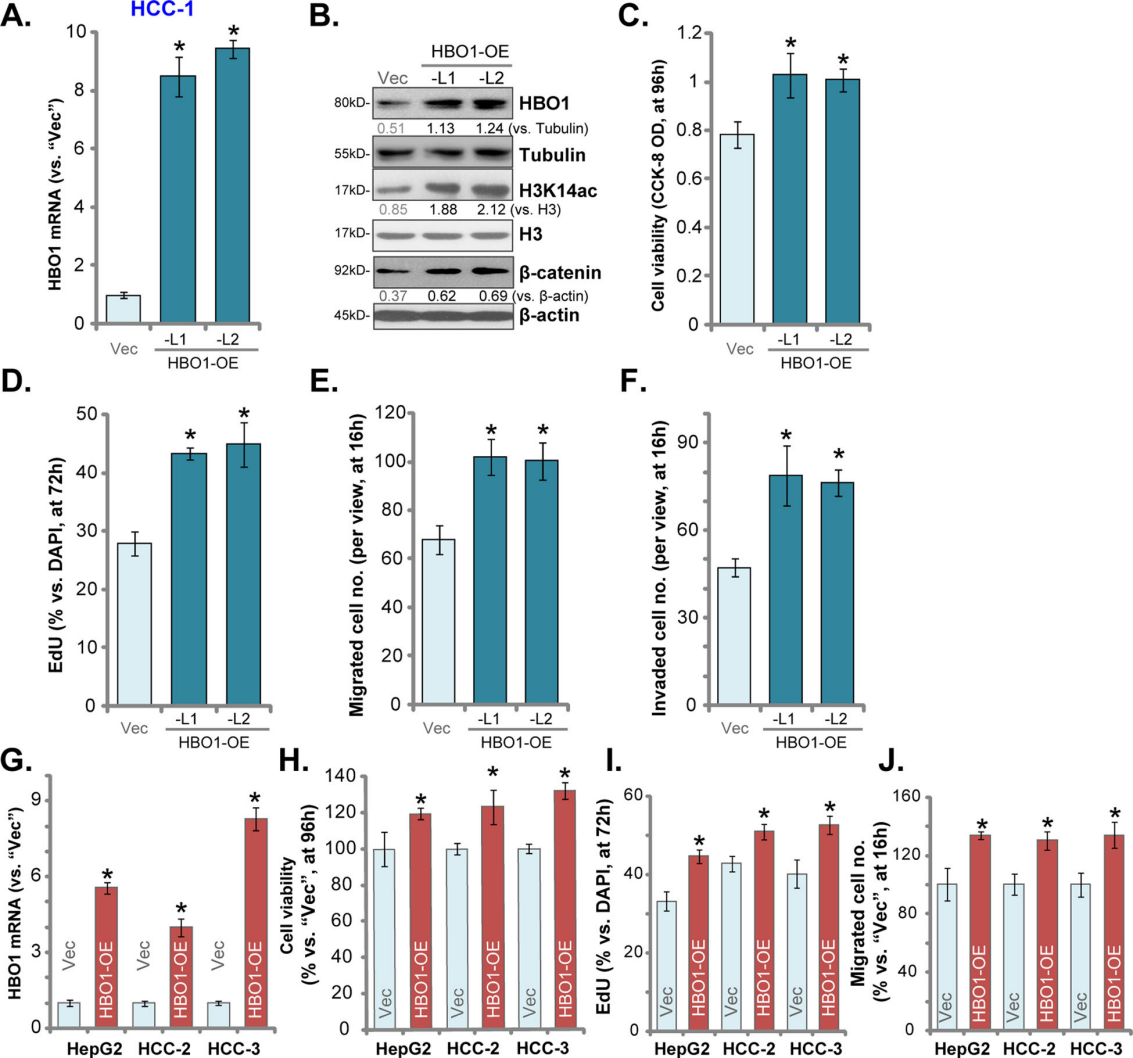
Ectopic overexpression of HBO1 promotes HCC cell progression in vitro. Stable primary human HCC cells (HCC-1/-2/-3) and HepG2 cells, with the HBO1-expression construct (“HBO1-OE”) or empty vector (“Vec”), were cultured for the applied time periods, expression of *HBO1* mRNA (**A** and **G**) and protein (**B**) was shown; Cellular functions, including cell viability (CCK-8 OD, **C** and **H**), proliferation (nuclear EdU incorporation assays, **D** and **I**), cell migration and invasion (**E**, **F** and **J**), were tested, with results quantified. Error bars indicate mean ± standard deviation (SD, *n* = 5). *P < 0.05 vs. cells with “Vec”. The experiments in this figure were repeated five times, and similar results were obtained.

The results have shown that HBO1 silencing or KO will result in significant inhibition of HCC cell progression. We proposed that forced overexpression of HBO1 should exert opposite functions. To test this hypothesis, a HBO1-expressing GV369 construct (with GFP) was transduced to HCC-1 primary cells. GFP-positive cells were sorted by FACS, and puromycin was added to select stable cells. Two stable cell lines, HBO1-OE-L1 and HBO1-OE-L2, were established. As compared to the vector control cells, *HBO1* mRNA levels increased over 8–10-folds in HBO1-OE HCC-1 cells (Fig. 5A), and HBO1 protein overexpression detected as well (Fig. 5B). H3K14 acetylation was enhanced in HBO1-overexpressed cells (Fig. 5B). *MYLK*, *VEGFR2*, *PBX3*, *CCR2*, *HOXA10,* and *FRZB* mRNAs were upregulated in HBO1-OE HCC-1 cells (Fig. S1C). β-catenin protein levels were increased as well (Fig. 5B).

Testing cell viability, by CCK-8 assays, confirmed that viability was enhanced in HBO1-OE HCC-1 cells (vs. control cells, Fig. 5C). Additional studies demonstrated that forced overexpression of HBO1 promoted HCC-1 cell proliferation by increasing nuclear EdU incorporation ratio (Fig. 5D). Furthermore, as compared to the vector control cells, in vitro cell migration (results quantified in Fig. 5E) and invasion (results quantified in Fig. 5F) were augmented in HBO1-OE cells.

In HepG2 cells, HCC-2 and HCC-3 primary cancer cells, stable expression of the HBO1-expressing GV369 construct (“HBO1-OE”) resulted in significant upregulation of *HBO1* mRNA (Fig. 5G). Functional studies demonstrated that HBO1 overexpression enhanced cell viability (CCK-8 OD, Fig. 5H), proliferation (by recording EdU-positive nuclei ratio, Fig. 5I) and migration (results quantified in Fig. 5J) in HepG2 and primary HCC cells.

In the primary HCCs derived from two other patients (HCC-4 and HCC-5), HBO1 protein expression was relatively low (vs. HCC-1 cells) (Fig. S3A). The application of the HBO1-expressing construct resulted in HBO1 overexpression in HCC-4 and HCC-5 cells (“HBO1-OE”, Fig. S3B). Ectopic overexpression of HBO1 augmented cell proliferation (by recording EdU-positive nuclei ratio, Fig. S3C) and migration (“Transwell” assays, results quantified in Fig. S3D) in HCC-4 and HCC-5 cells as well. These results together showed that ectopic overexpression of HBO1 promoted HCC cell proliferation, migration, and invasion, further supporting the role of HBO1 in HCC cell progression.

### HBO1 KO inhibits HCC xenograft growth in SCID mice

**Fig. 6:**
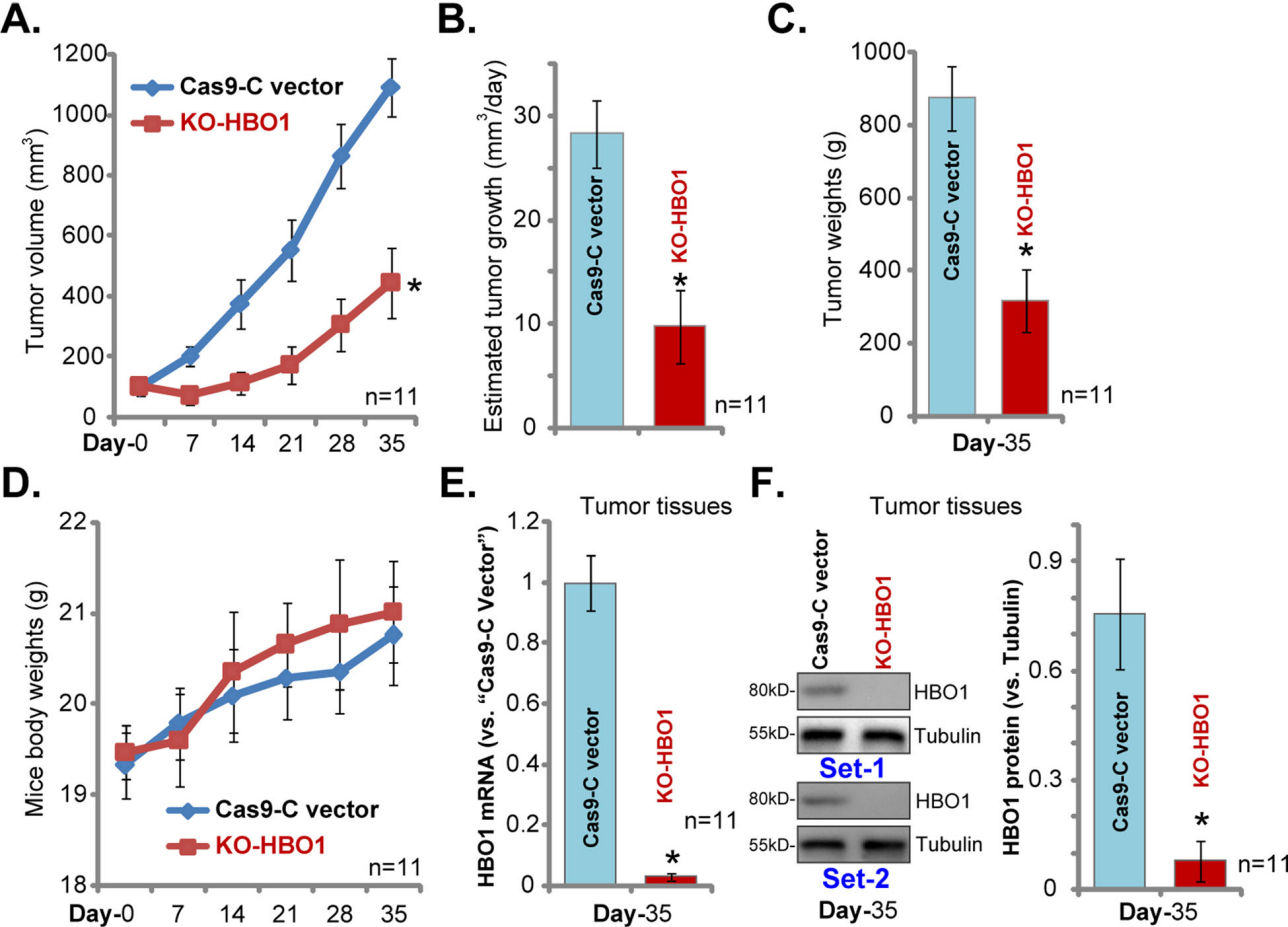
HBO1 KO inhibits HCC xenograft growth in SCID mice. Primary HCC1 cells, bearing CRISPR/Cas9 HBO1-KO construct (“KO-HBO1”) or the empty vector (“Cas9-C vector”), were *s.c*. inoculated to the SCID mice (with 11 mice per group). Recordings were started when the volume of each tumor close to 100 mm^3^ (“Day-0”). Tumor volumes (**A**) and mice body weights (**D**) were recorded every seven days for a total of 35 days; Estimated daily tumor growth was calculated as described (**B**); At Day-35 all tumors were isolated and weighted individually (**C**). The tumor lysates were subjected to qPCR (**E**) and Western blotting (**F**) assays. Error bars indicate mean ± standard deviation (SD, *n* = 5). *P < 0.05 vs. “Cas9-C vector” tumors.

At last, we tested the potential effect of HBO1 on HCC cell growth in vivo. Primary HCC1 cells, with the CRISPR/Cas9 HBO1-KO construct (“KO-HBO1”) or the empty vector (“Cas9-C vector”) (see Fig. 4), were inoculated via *s.c*. injection to SCID mice. Recordings were started when the volume of each xenograft tumor close to 100 mm^3^ (“Day-0”). As demonstrated, KO-HBO1 HCC1 xenografts grew significantly slower than the control xenografts (Fig. 6A). Volumes of KO-HBO1 HCC1 xenografts were significantly lower than those of control tumors (Fig. 6A). When calculating the estimated daily tumor growth via the formula: (Tumor volume at Day-35 − Tumor volume at Day-0)/35, we show that HCC1 xenograft growth in vivo was significantly inhibited with HBO1 KO (Fig. 6B).

At Day-35 all tumors were isolated and weighted individually. Results demonstrated that KO-HBO1 HCC1 xenografts weighted much lower than Cas9-C vector control xenografts (Fig. 6C). On the contrary, the mice body weights were not significantly different between the two groups (Fig. 6D). The xenograft tumor tissues were also analyzed through qPCR (Fig. 6E) and Western blotting (Fig. 6F), and results confirmed HBO1 depletion in the KO-HBO1 HCC1 xenograft tissues (Fig. 6E, F). Collectively, these results demonstrated that HBO1 KO inhibited HCC xenograft growth in SCID mice.

## Discussion

Recent studies have reported gene amplification and protein overexpression of HBO1 in human malignancies, which is linked to tumorigenesis and cancer progression. Studies have shown that HBO1 overexpression promoted bladder and breast cancer cell proliferation and tumorigenesis^16,23,46^. In pancreatic cancer cells, HBO1 expression contributed to gemcitabine resistance^47^. Wang et al., reported that HBO1 overexpression is associated with poor prognosis in gastric cancer^48^. A very recent study has demonstrated that HBO1 is required for maintenance of leukemia stem cells (LSC) in acute myeloid leukemia (AML)^21^. Interestingly, Kahali et al., demonstrated that HBO1 activity can induce the expression of anti-cancer genes such as Brahma^49^. It is therefore important to examine HBO1 expression and its functions in specific types of cancers with different genetic backgrounds^10^.

Because of the direct interaction between HBO1 and DNA pre-replication complex proteins, HBO1 is proposed to be required for DNA replication and cell proliferation^50^. However, Kueh et al., have suggested that cancer cell lines with an established transcription profile were relatively insensitive to HBO1 depletion, and did express genes required for cell proliferation^25^. We therefore tested HBO1’s function mainly in primary HCC cells. The results of the present study implied that HBO1 is an important oncogenic gene and therapeutic target of HCC. *HBO1* mRNA and protein expression is elevated in human HCC tissues as well as in established and primary human HCC cells. While its expression is low in liver tissues and hepatocytes. In HepG2 and primary human HCC cells, HBO1 silencing, by targeted shRNA, potently inhibited cell viability, proliferation, migration and invasion, while provoking apoptosis. Additionally, CRISPR/Cas9-induced HBO1 KO inhibited HCC cell progression and induced apoptosis activation. Conversely, forced overexpression of HBO1, through a lentiviral construct, promoted HCC cell proliferation, migration, and invasion. In vivo, the growth of xenografts bearing HBO1-KO HCC cells was largely inhibited in SCID mice. These results suggest that targeting HBO1 could be a novel and valuable strategy to inhibit HCC growth.

Studies have proposed different mechanisms of HBO1-induced cancer progression. Quintela et al., reported that HBO1 directly acetylated histone H4 to promote expression of a key oncogene YAP1, required for mechano-transduction and membrane elasticity in ovarian cancer cells^19^. Chen et al., found that HBO1 can activate Wnt/β-catenin signaling pathway to promote bladder cancer cell progression^23^. Iizuka and colleagues demonstrated that HBO1 destabilized estrogen receptor α by ubiquitination to promote breast cancer cell growth^51^. Future studies will be needed to understand the underlying mechanisms of HBO1 in promoting HCC cell progression.

The majority of HCC are in Asia-Pacific counties, yet rising incidence has been reported in the Western World, possibly due to increasing non-alcoholic fatty liver disease (NAFLD)^3,4^. For clinical practices, current therapeutic options for HCC include liver resection, immunotherapy, and chemotherapy drugs^3,4^. For advanced HCC patients, a multitarget kinase inhibitor sorafenib is possibly the only available systemic therapy, which can slightly increase the survival of certain unresectable HCC patients^52,53^. Since the approve of sorafenib, many other targeted therapies, including sunitinib, tivantinib, brivanib, erlotinib, linifanib, and bevacizumab have been tested, but showing no meaningful improvement in treatment of HCC^54^. Song et al., found that polo-like kinase 1 (Plk1)-induced phosphorylation of HBO1 transcriptionally increased expression of cFos and multidrug resistance 1 (MDR1), essential for gemcitabine’s resistance in pancreatic cancer cells^47^. Future studies will be needed to explore the potential role of HBO1 in overwhelming chemoresistance in HCC^7,55,56^. These results suggest HBO1 overexpression is important for HCC cell progression in vitro and in vivo. It could be a promising oncogenic gene and therapeutic target of HCC.

## Supplementary information

Supplementary Figures

## References

[1] Siegel RL, Miller KD, Jemal A (2020). Cancer statistics, 2020. Cancer J. Clin..

[2] Siegel RL, Miller KD, Jemal A (2019). Cancer statistics, 2019. Cancer J. Clin..

[3] Singal AG, Lampertico P, Nahon P (2020). Epidemiology and surveillance for hepatocellular carcinoma: new trends. J. Hepatol..

[4] Akinyemiju T, Global Burden of Disease Liver Cancer, Collaboration (2017). The burden of primary liver cancer and underlying etiologies from 1990 to 2015 at the Global, regional, and National Level: results from the Global burden of Disease Study 2015. JAMA Oncol..

[5] Yang JD, Roberts LR (2010). Hepatocellular carcinoma: a global view. Nat. Rev. Gastroenterol. Hepatol..

[6] Qiu L, Tang Q, Li G, Chen K (2017). Long non-coding RNAs as biomarkers and therapeutic targets: recent insights into hepatocellular carcinoma. Life Sci..

[7] Stotz M (2015). Molecular targeted therapies in hepatocellular carcinoma: past, present and future. Anticancer Res..

[8] Wang Q (2010). Acetylation of metabolic enzymes coordinates carbon source utilization and metabolic flux. Science.

[9] Zhao S (2010). Regulation of cellular metabolism by protein lysine acetylation. Science.

[10] Lan R, Wang Q (2020). Deciphering structure, function and mechanism of lysine acetyltransferase HBO1 in protein acetylation, transcription regulation, DNA replication and its oncogenic properties in cancer. Cell Mol. Life Sci..

[11] Marmorstein R, Zhou MM (2014). Writers and readers of histone acetylation: structure, mechanism, and inhibition. Cold Spring Harb. Perspect. Biol..

[12] Allfrey VG, Faulkner R, Mirsky AE (1964). Acetylation and methylation of histones and their possible role in the regulation Of Rna synthesis. Proc. Natl Acad. Sci. USA.

[13] Zhao J, Gray SG, Greene CM, Lawless MW (2019). Unmasking the pathological and therapeutic potential of histone deacetylases for liver cancer. Expert Rev. Gastroenterol. Hepatol..

[14] Wu ZQ, Liu X (2008). Role for Plk1 phosphorylation of Hbo1 in regulation of replication licensing. Proc. Natl Acad. Sci. USA.

[15] Doyon Y (2006). ING tumor suppressor proteins are critical regulators of chromatin acetylation required for genome expression and perpetuation. Mol. Cell..

[16] Iizuka M (2009). Histone acetyltransferase Hbo1: catalytic activity, cellular abundance, and links to primary cancers. Gene.

[17] Kim MS (2015). The histone acetyltransferase Myst2 regulates Nanog expression, and is involved in maintaining pluripotency and self-renewal of embryonic stem cells. FEBS Lett..

[18] Sapountzi V, Cote J (2011). MYST-family histone acetyltransferases: beyond chromatin. Cell Mol. Life Sci..

[19] Quintela M (2019). HBO1 directs histone H4 specific acetylation, potentiating mechano-transduction pathways and membrane elasticity in ovarian cancer cells. Nanomedicine.

[20] Ohzeki J (2016). KAT7/HBO1/MYST2 regulates CENP-A chromatin assembly by antagonizing Suv39h1-mediated centromere inactivation. Dev. Cell.

[21] MacPherson L (2020). HBO1 is required for the maintenance of leukaemia stem cells. Nature.

[22] Gao YY (2021). The histone acetyltransferase HBO1 functions as a novel oncogenic gene in osteosarcoma. Theranostics.

[23] Chen Z (2018). HBO1 promotes cell proliferation in bladder cancer via activation of Wnt/beta-catenin signaling. Mol. Carcinog..

[24] Taniue K (2020). UHRF1-KAT7-mediated regulation of TUSC3 expression via histone methylation/acetylation is critical for the proliferation of colon cancer cells. Oncogene.

[25] Kueh, A. J. et al. HBO1 (KAT7) does not have an essential role in cell proliferation, DNA replication, or histone 4 acetylation in human cells. Mol. Cell Biol. **40**, e00506–19 (2020). 10.1128/MCB.00506-19 PMC6996278 31767635

[26] Chen MB (2016). KU-0060648 inhibits hepatocellular carcinoma cells through DNA-PKcs-dependent and DNA-PKcs-independent mechanisms. Oncotarget.

[27] Cheng L (2017). Identification of DNA-PKcs as a primary resistance factor of TIC10 in hepatocellular carcinoma cells. Oncotarget.

[28] Bai JY (2021). Requirement of Galphai1 and Galphai3 in interleukin-4-induced signaling, macrophage M2 polarization and allergic asthma response. Theranostics.

[29] Xu M (2020). The therapeutic value of SC66 in human renal cell carcinoma cells. Cell Death Dis..

[30] Zheng B (2015). Pre-clinical evaluation of AZD-2014, a novel mTORC1/2 dual inhibitor, against renal cell carcinoma. Cancer Lett..

[31] Brooks MM, Neelam S, Fudala R, Gryczynski I, Cammarata PR (2013). Lenticular mitoprotection. Part A: monitoring mitochondrial depolarization with JC-1 and artifactual fluorescence by the glycogen synthase kinase-3beta inhibitor, SB216763. Mol. Vis..

[32] Zhang YM (2015). Requirement of Galphai1/3-Gab1 signaling complex for keratinocyte growth factor-induced PI3K-AKT-mTORC1 activation. J. Invest. Dermatol..

[33] Chandrashekar DS (2017). UALCAN: a portal for facilitating tumor subgroup gene expression and survival analyses. Neoplasia.

[34] Yan MS (2018). Histone acetyltransferase 7 (KAT7)-dependent intragenic histone acetylation regulates endothelial cell gene regulation. J. Biol. Chem..

[35] Zhuang H, Cao G, Kou C, Liu T (2018). CCL2/CCR2 axis induces hepatocellular carcinoma invasion and epithelial-mesenchymal transition in vitro through activation of the Hedgehog pathway. Oncol. Rep..

[36] Li X (2017). Targeting of tumour-infiltrating macrophages via CCL2/CCR2 signalling as a therapeutic strategy against hepatocellular carcinoma. Gut.

[37] Lin J (2018). MYLK promotes hepatocellular carcinoma progression through regulating cytoskeleton to enhance epithelial-mesenchymal transition. Clin. Exp. Med..

[38] Saraswati S, Alhaider A, Abdelgadir AM, Tanwer P, Korashy HM (2019). Phloretin attenuates STAT-3 activity and overcomes sorafenib resistance targeting SHP-1-mediated inhibition of STAT3 and Akt/VEGFR2 pathway in hepatocellular carcinoma. Cell Commun. Signal..

[39] Xiang Q (2014). Cabozantinib suppresses tumor growth and metastasis in hepatocellular carcinoma by a dual blockade of VEGFR2 and MET. Clin. Cancer Res..

[40] Zhang Y (2019). HOXA10 knockdown inhibits proliferation, induces cell cycle arrest and apoptosis in hepatocellular carcinoma cells through HDAC1. Cancer Manage Res..

[41] Xiao ZD (2014). miR-218 modulate hepatocellular carcinoma cell proliferation through PTEN/AKT/PI3K pathway and HoxA10. Int. J. Clin. Exp. Pathol..

[42] Wang M, Lv G, Jiang C, Xie S, Wang G (2019). miR-302a inhibits human HepG2 and SMMC-7721 cells proliferation and promotes apoptosis by targeting MAP3K2 and PBX3. Sci. Rep..

[43] Han H (2015). PBX3 is targeted by multiple miRNAs and is essential for liver tumour-initiating cells. Nat. Commun..

[44] Huang J, Hu W, Lin X, Wang X, Jin K (2015). FRZB up-regulated in hepatocellular carcinoma bone metastasis. Int. J. Clin. Exp. Pathol..

[45] Au, Y. Z., et al. KAT7 is a genetic vulnerability of acute myeloid leukemias driven by MLL rearrangements. Leukemia 35, 1012–1022 (2020). 10.1038/s41375-020-1001-z PMC7610570 32764680

[46] Hu X (2009). Genetic alterations and oncogenic pathways associated with breast cancer subtypes. Mol. Cancer Res..

[47] Song B (2013). Plk1 phosphorylation of orc2 and hbo1 contributes to gemcitabine resistance in pancreatic cancer. Mol. Cancer Ther..

[48] Wang Y (2019). High-expression HBO1 predicts poor prognosis in gastric cancer. Am. J. Clin. Pathol..

[49] Kahali B (2014). Identifying targets for the restoration and reactivation of BRM. Oncogene.

[50] Alabert C, Groth A (2012). Chromatin replication and epigenome maintenance. Nat. Rev. Mol. Cell Biol..

[51] Iizuka M (2013). Histone acetyltransferase Hbo1 destabilizes estrogen receptor alpha by ubiquitination and modulates proliferation of breast cancers. Cancer Sci..

[52] Cheng AL (2009). Efficacy and safety of sorafenib in patients in the Asia-Pacific region with advanced hepatocellular carcinoma: a phase III randomised, double-blind, placebo-controlled trial. Lancet Oncol..

[53] Llovet JM (2008). Sorafenib in advanced hepatocellular carcinoma. N. Engl. J. Med..

[54] Tella, S. H., Kommalapati, A. & Mahipal, A. Systemic therapy for advanced hepatocellular carcinoma: targeted therapies. *Chin. Clin. Oncol.* **10**, 10 (2020). 10.21037/cco-20-117 32434345

[55] Singh S, Singh PP, Roberts LR, Sanchez W (2014). Chemopreventive strategies in hepatocellular carcinoma. Nat. Rev. Gastroenterol. Hepatol..

[56] Llovet JM, Bruix J (2008). Molecular targeted therapies in hepatocellular carcinoma. Hepatology.

