# Supplementary material for: HBO1 overexpression is important for hepatocellular carcinoma cell growth
Source: Cell Death Dis. 2021 May 26;12(6):549. doi: 10.1038/s41419-021-03818-1 (PMC8155027; doi:10.1038/s41419-021-03818-1)
Supplement: Supplementary file 1 — Supplementary Figures [file 41419_2021_3818_MOESM1_ESM.pdf]

Figure S1

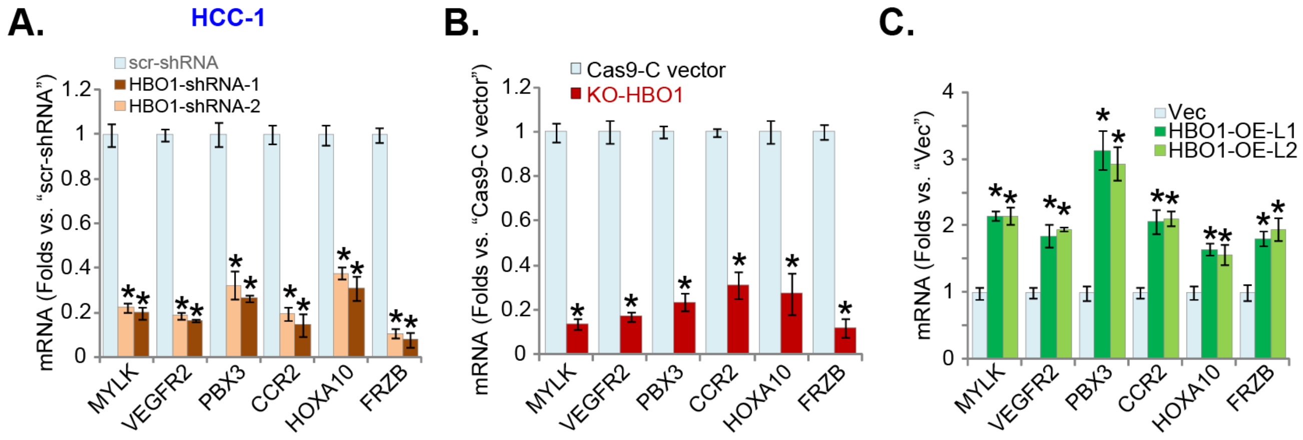

Figure S1. Relative expression of listed mRNAs in HCC-1 cells with applied genetic modifications was tested by qPCR assays, and results were quantified (**A**-**C**). Error bars indicate mean ± standard deviation (SD, n=5). The experiments in this figure were repeated five times, and similar results were obtained. * ***P***<0.05 vs. "scr-shRNA" cells (**A**), "Cas9-C vector" cells (**B**) or "Vec" cells (**C**).

Figure S2

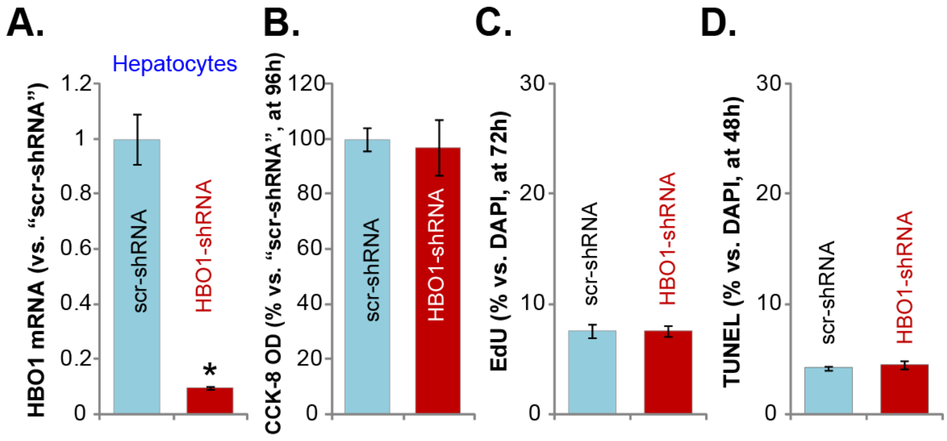

Figure S2. The primary human hepatocytes with scramble control shRNA ("scr-shRNA") or HBO1 shRNA-2 ("HBO1-shRNA") were cultured for applied time periods, expression of *HBO1* mRNA was shown (**A**); Cell viability (CCK-8 OD, **B**), proliferation (nuclear EdU incorporation, **C**) and apoptosis (nuclear TUNEL ratio, **D**) were tested. Error bars indicate mean ± standard deviation (SD, n=5). The experiments in this figure were repeated five times, and similar results were obtained. * ***P***<0.05 vs. "scr-shRNA" cells (**A**).

Figure S3

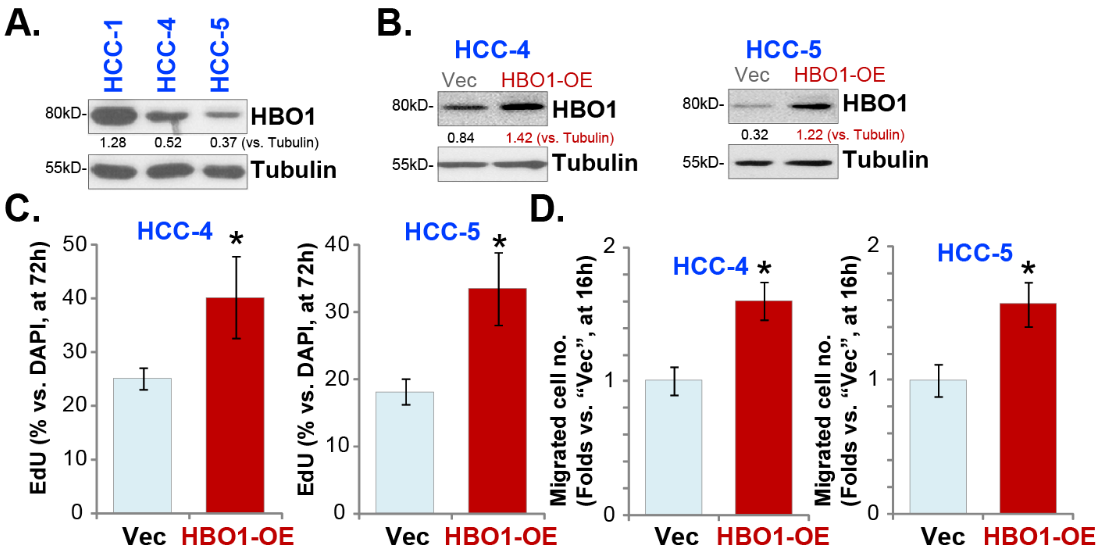

Figure S3. HBO1 and Tubulin protein expression in listed primary HCC cells (HCC-1/HCC-4/HCC-5) was shown (**A**). Expression of HBO1 and Tubulin in HCC-4/HCC-5 cells with the HBO1-expression construct ("HBO1-OE") or empty vector ("Vec") was shown (**B**); Cells were further cultured for applied time periods, cell proliferation (by recording nuclear EdU ratio, **C**) and cell migration ("Transwell" assays, **D**) were tested, with results quantified. Error bars indicate mean ± standard deviation (SD, n=5). The experiments in this figure were repeated five times, and similar results were obtained. * ***P***<0.05 vs. "Vec" cells (**C** and **D**).
